# An UHPLC-HRMS-Based Untargeted Metabolomics Approach to Explore the Effects of Bacterial Endophyte Co-Culture on *Alkanna tinctoria* (L.) Tausch Cell Suspension Metabolome

**DOI:** 10.3390/microorganisms13071601

**Published:** 2025-07-07

**Authors:** Elodie Bossard, Adrien Cousy, Antonio Grondin, Nikolaos Tsafantakis, Angélique Rat, Nektarios Aligiannis, Anne Willems, Laetitia Cattuzzato, Thien Nguyen, Nikolas Fokialakis

**Affiliations:** 1 Department of Pharmacy, National and Kapodistrian University of Athens, 15771 Athens, Greecealigiannis@pharm.uoa.gr (N.A.); 2 Pierre Fabre Dermo-Cosmetic & Personal Care (PFDC&PC), 31100 Toulouse, France; 3 Herbal Products Laboratory, Green Mission Department, Pierre Fabre Research Institute, 31035 Toulouse, France; antonio.grondin@pierre-fabre.com; 4 Laboratory of Microbiology, Faculty of Sciences, Ghent University, 9000 Ghent, Belgiumanne.willems@ugent.be (A.W.)

**Keywords:** bacterial endophytes, plant–endophyte relationships, plant cell culture, co-culture, hydroxynaphthoquinones

## Abstract

Colonization of plant tissues by bacterial endophytes might lead to qualitative and quantitative changes in secondary metabolites (SMs). In this work, in vitro co-culture experiments were performed using cell suspensions of the medicinal plant *Alkanna tinctoria* and eight of its bacterial endophytes. An untargeted metabolomics approach using Ultra-High-Performance Liquid Chromatography High-Resolution Mass Spectrometry (UHPLC-HRMS) was employed to investigate plant–microbe interactions. Hierarchical clustering analysis and principal component analysis highlighted significant modifications of specific regulation patterns in SM production, caused by bacterial endophytes. The annotation step lead to the identification of 32 stimulated compounds in *A. tinctoria* cell suspensions. Among them, 3′-hydroxy-14-hydroxyshikonofuran H (**5**), 8′-decarboxy-rosmarinic acid (**18**), 8‴-decarboxy-salvianolic B (**23**), 8″-8‴-didecarboxy-salvianolic acid B (**26**) were putatively identified for the first time. Our findings highlight that employing selected microbial inoculants under controlled conditions can be an effective strategy for enhancing or stimulating the production of specific high-value metabolites.

## 1. Introduction

In recent years, scientists are no longer considering plants as exclusively plant entities but rather as dynamic living organism systems. The term plant holobiont has emerged, describing the plant and all the microorganisms that live in and on it as a single complex entity [1]. Symbiotic interactions between the plant and these associated microorganisms are continuously established, leading to dynamic changes in the genome, metabolism, and signaling networks of the partners [2]. Among the plant-associated microorganisms, bacterial endophytes play an overall essential role in the plant’s balance. They colonize all plant organs and tissues (e.g., flowers, leaves, roots, seeds, and stems) and use the plant’s internal environment (i.e., the endosphere) as a unique niche to protect themselves from aggressive external environments [3]. In return, bacterial endophytes may benefit the plants by promoting their growth and enhancing their resistance toward various pathogens and environmental stress [4].

Earlier, it was also reported that bacterial endophytes significantly induce the production of secondary metabolites with important biological effects by their host plant through intimate molecular communications. Indeed, some metabolites are not only produced by a single organism, but might be generated by a plant in combination with associated bacteria [4]. Interestingly, the induction of secondary metabolite production by endophytes might be a much more widespread phenomenon in aromatic and medicinal plants [4,5]. For instance, a bacterial endophyte isolated from the roots of *Panax ginseng* significantly induced an important accumulation of Ginsenoside by its valuable medicinal herb host [6]. Furthermore, differences in the medicinal properties may be explained by the presence of distinct bacterial communities in different plant species [4].

*Alkanna tinctoria* (L.) Tausch is an important medicinal plant particularly known for accumulating in the external layer of its roots several bioactive compounds, mainly characterized by a hydroxynaphthoquinone structure: Alkannin/Shikonin and their ester derivatives (HNQs). This Mediterranean medicinal plant belongs to the Boraginaceae family with a limited distribution in southern Europe [7]. In traditional medicine, preparations made with its roots were commonly used to treat wounds, burns, and ulcers [8]. In the past decades, several in vitro and in vivo studies have provided evidence of the medicinal properties of its bioactive compounds. In detail, HNQs present anti-inflammatory [9], antimicrobial [10], antitumor activities [11,12,13] and especially wound healing properties [14].

According to a recent study, more than one hundred distinct phylotypes of bacterial endophytes were isolated from the roots of *A. tinctoria*. These microorganisms associated with its host plant demonstrate a potential plant growth promotion and might stimulate the production of its bioactive secondary metabolites [15]. The relationship between bacterial endophytes and their host plants remains poorly understood. Therefore, *A. tinctoria*, along with the recent discoveries about its endo-microbiome, represents a valuable model for investigating this relationship and the metabolomic changes linked to this association.

In this work, *A. tinctoria*–bacterial endophyte interactions were reproduced in vitro by using biotechnology approaches [16]. The interaction between *A. tinctoria* cell suspension and eight of its bacterial endophytes was investigated through an Ultra-High-Performance Liquid Chromatography-High-Resolution Mass Spectrometry (UHPLC-HRMS) untargeted metabolomics approach. The main bacterial endophyte-induced metabolites were putatively identified and classified using UHPLC-HRMS-based hierarchical clustering analysis. This approach may provide a successful understanding of bacterial endophyte–medicinal plant interaction and may also facilitate the discovery of unknown compounds.

## 2. Materials and Methods

**Figure 1:**
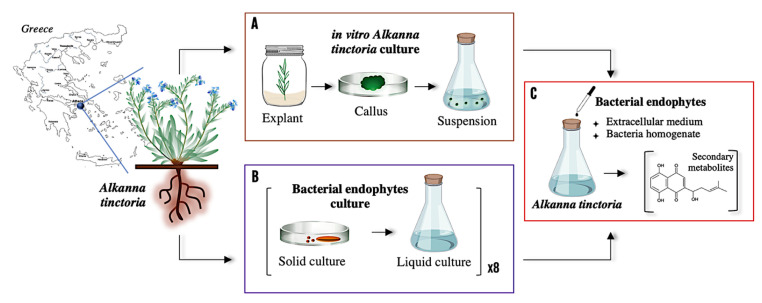
Schematic representation of the investigation including (**A**) in vitro culture of *A. tinctoria*, (**B**) in vitro culture of eight bacterial endophytes isolated from the roots of *A. tinctoria* (i.e., *Chitinophaga* sp., *Xanthomonas* sp., *Pseudomonas* sp., *Micromonospora* sp., *Allorhizobium* sp., *Rhizobium* sp., *Brevibacillus* sp., *Brevibacterium* sp.), and (**C**) in vitro screening of the eight bacterial endophytes on *A. tinctoria* cell suspension.

To investigate the relationship between bacterial endophytes and their host plant, co-cultures of eight different strains isolated from wild roots and in vitro cell suspension of *A. tinctoria* were conducted following the steps summarized in Figure 1.

### 2.1. Chemicals

The extraction and development solvents including ethyl acetate (EtoAc) and ethanol absolute (EtOH abs) were purchased from Merck (Darmstadt, Germany). LC/MS grade formic acid as well as dimethyl sulfoxide (DMSO) were supplied from Sigma Aldrich (St Quentin Fallavier, France). LC/MS grade solvents such as methanol (MeOH) and acetonitrile (ACN) were obtained from Fisher Scientific (Illkrich, France) and CarloErba (Val de Reuil, France), respectively. Ultra-pure 18 MΩ water was obtained from a Milli-Q water purification system (Merck Millipore, Fontenay sous bois, France).

### 2.2. Biological Material

#### 2.2.1. *A. tinctoria* Cells Suspension

Callus culture of *A. tinctoria* was initiated from in vitro explants provided by the Hellenic agricultural organization (Thessaloniki, Greece). Callus was grown in Gamborg (B5) medium supplemented with 1 mg/L of indole-3-acetic acid (IAA), 2 mg/L 6-benzylaminopurine (BAP), 3% of sucrose, and solidified with 0.8% agar (KOBE I). The medium pH was adjusted to 6.0 before autoclaving (121 °C for 20 min). The culture was incubated at 27 °C in the complete darkness and subcultured every 4 weeks. Suspension cultures were established from 4-week-old well-grown callus cultures. About 10 g of fresh friable callus was immerged in 50 mL (250 mL flasks) of B5 medium supplemented with 1 mg/L of IAA, 2 mg/L BAP, 3% of sucrose at a shaking rate of 130 rpm, and a temperature of 27 °C. The pH of the media was adjusted to 5.8 before autoclaving (121 °C for 20 min). The cell suspension culture was subcultured every 14 days at a concentration of 90 g/L [7].

#### 2.2.2. Bacterial Endophytes

Eight bacterial endophytes isolated from the roots of wild *A. tinctoria*, previously identified and characterized by Rat et al. [15], were provided by the Laboratory of Microbiology of Ghent University (Table 1). Among the selected bacterial endophytes, an effective induction of HNQs production on hairy roots by five strains were already observed by Rat et al. (i.e., *Chitinophaga* sp., *Xanthomonas* sp., *Pseudomonas* sp., *Micromonospora* sp., *Allorhizobium* sp.) [15]. During this study, bacterial endophyte cultures were maintained on a solid R2A medium (pH 7.2) at 4 °C, except the genus *Micromonospora* sp. which was grown in liquid R2B medium (pH 7.2) at 28 °C in a 250 mL shake flask at 120 rpm (KS-15 shaker, Edmud Bühler, Bodelshausen, Germany), and not grown on solid medium [15].

### 2.3. Co-Culture Experimental Set Up

An in vitro culture of the bacterial endophyte and host plant cell suspension may be defined as “co-culture” when bacterial components such as bacterial endophyte cells, bacterial cellular elements and/or bacterial extracellular medium are involved. In our study, bacterial homogenates prepared from bacterial endophyte cells and extracellular medium were used for the co-culture.

#### 2.3.1. Preparation of Bacterial Endophyte Components

The bacterial endophyte components were prepared by growing the bacteria in liquid R2 broth medium (Carl ROTH, Lauterbourg, France) (pH 7.2) at 28 °C in shake flasks of 250 mL at 120 rpm (Stirrer Edmud Bühler KS-15) for 4 h, reaching a maximum optical density (OD) value of 1. The OD value was monitored with a spectrophotometer (BioRad smartspec Plus, Hercules, CA, USA) at 600 nm. Bacteria cells were harvested from the liquid culture (with an OD of 1) in R2 broth medium by centrifugation at 4000 rpm at 4 °C during 15 min. The cell mass was resuspended in distilled water (5 g in 100 mL) and autoclaved at 121 °C for 20 min. The bacterial endophyte culture supernatant (used medium) were obtained by filter-sterilization of the culture broth (sterile 0.2 µm filter; cellulose acetate; Ministart, Sartorius, Bernolsheim, France). The sterilized used medium was then lyophilized and resuspended in distilled water at a concentration of 10 g/L [16].

#### 2.3.2. Co-Culture Experiment

Either the biomass homogenate (BaH), or the bacterial endophyte culture supernatant (ECM) of each bacterial cell line was inoculated into *A. tinctoria* cells suspension, freshly subcultured at 200 g/L, in glass tubes at 0.04% and 4%, respectively (*v*/*v*; 1.6–160 µL broth in 4 mL) (Table 1). The co-cultures were incubated at 27 °C in the complete darkness at a shaking rate of 50 rpm. For comparative purposes, simultaneous cell suspension cultures were carried out without inoculation of bacterial components and with the phytohormone methyl jasmonate at 50 µM as positive control, following the culture parameters described above. All conditions were performed in triplicates [16].

### 2.4. Extraction of Secondary Metabolites

Six-day-old co-cultures were collected and lyophilized. A quantity of 60 mg of dry suspension was subjected to an ultrasound-assisted extraction using successively 1.8 mL of EtoAc and EtOH (liquid solid ratio 1:30) at room temperature for 20 min twice. The mixture was centrifuged at 14,000 rpm for 5 min at 4 °C (Mikro 220R, hettich zentrigugen, Föhrenstr, Germany). The supernatant obtained was then evaporated using a centrifugal concentrator (Genevac HT-4x). Finally, extracts were resuspended in DMSO at a concentration of 20 mg/mL (stock solutions). For UHPLC-MS analysis, stock solutions were diluted at a concentration of 2 mg/mL with methanol UHPLC grade (15 µL SS + 135 µL MeOH).

### 2.5. UHPLC-HRMS Analysis

#### 2.5.1. LC System and Chromatographic Conditions

An Acquity UPLC-system™ (Waters SAS, Saint Quentin en Yvelines, Montigny-le-Bretonneux, France) including, a quaternary high-pressure gradient pump, an automatic sample injector, a two-column thermostated oven and a photodiode array detector were used. Chromatographic separation was achieved on an Acquity BEH Shield RP C18, 1.7 µm, 50 × 2.1 mm column with an Acquity BEH C18, 1.7 µm, 20 × 2.1 mm as a guard column (Saint Quentin en Yvelines, Montigny-le-Bretonneux, France). Mobile phase A consisted of in-house deionized water containing 0.1% formic acid, and mobile phases B and C consisted of ACN and MeOH respectively. A H2O/ACN/MeOH (1:2:7, *v*/*v*/*v*) mixture was used as the injector wash solution. An 8 min method was applied at a flow rate of 0.4 mL/min and varying conditions as described: the initial gradient condition was 90% A—10% B linearly changed to 2%A—98% B over 6 min with a curve 7 and held until 6.10; changed to 100% C and held from 6.15 to 6.50; changed to 2% A—98% B and held from 6.55 until 6.95; turned back to the initial condition from 7.00 until 8.00 with a flow rate of 0.5 mL/min from 7.05 to 7.90. The column temperature was adjusted at 35 °C. The injection volume was 1 µL. The UV detector was set on a range of 200–500 nm with a sampling rate of 5 points s^‒1^ and a resolution of 1.2 nm.

#### 2.5.2. High-Resolution Mass Spectrometry Conditions

HRMS experiments were recorded on a Synapt G2Si with Masslynx v4.1 software (Waters SAS, Saint Quentin en Yvelines, Montigny-le-Bretonneux, France) equipped with an ESI ion source. The instrument was calibrated using a sodium formate solution as the calibration standard as suggested by the manufacturer, and this calibration allowed for mass accuracies of <5 ppm. To ensure stable and precise scanning, leucine enkephalin (1 ng mL^−1^, 10 µL min^−1^) was used as reference compound and sprayed into the source every 20 s (positive ions *m*/*z* 556.2771 and 278.1141 and negative ions 554.2615 and 236.1035); correction was not applied while acquiring data and was performed at the reprocessing steps. The MS source temperature was set at 125 °C and the desolvation temperature was set at 500 °C. Nitrogen was used as the dry gas: the desolvation gas flow rate was set at 1000 L h^−1^; the cone gas flow was maintained at 50 L h^−1^. In both positive and negative modes, capillary and cone voltages were set at 0.5 kV and 50 V, respectively. All data were collected in continuum mode in resolution mode (resolving power in excess of 20,000) with the ion acquisition time of 0.1 s and with a mass range from *m*/*z* 100 to 1600. MSe experiments alternating low and high collision energy scans were performed with collision settings of 4 V and a ramp from 15 to 50 V for the low energy and high energy scans, respectively. Data-Dependent Acquisitions (DDAs) were performed by triggering MS/MS experiments for the four most intense peaks observed during the survey scan. Collision energy conditions were as follows: survey scan collision settings of 4 V; ramp from 30 to 50 V for the MS/MS experiments.

### 2.6. UHPLC-HRMS Data Processing

Raw data were noise-reduced before transferring them to servers for reprocessing with Waters Compression and Archival Tool v 1.10 (Waters SAS, Saint Quentin en Yvelines, France). MSe experiments were reprocessed for untargeted metabolomic analysis with Progenesis QI (Waters SAS, Saint Quentin en Yvelines, France) to obtain CSV tables for further statistical analyses (see Section 2.7). Annotation was performed using both MSe and MS/MS data with MassLynx 4.1 and UNIFI (v1.8.2) (Waters SAS, Saint Quentin en Yvelines, France). This step was performed by comparing the experimental results obtained (accurate masses and MS/MS spectra) with structural databases (Dictionary of Natural Products (Chapman Hall. Dictionary of Natural Products, http://dnp.chemnetbase.com/ (accessed on 14 October 2023), Atlas library, Chemspider) and with data from the literature.

### 2.7. Statistical Analysis

All statistical models and graphs, such as principal component analysis (PCA), Heatmap, and Boxplot, were computed using the open-source software RStudio version 1.2.5033 (2009–2019 RStudio, Inc., Boston, MA, USA) and the following packages: “FactoMineR” (R package version 2.3) [17] and “ggplot2” (R package version 3.3.2) [18]. The ANOVA and Tukey’s test were performed using the same software and the following package: “agricolae” [19].

## 3. Results

### 3.1. Total Suspension Dry Weight

**Figure 2:**
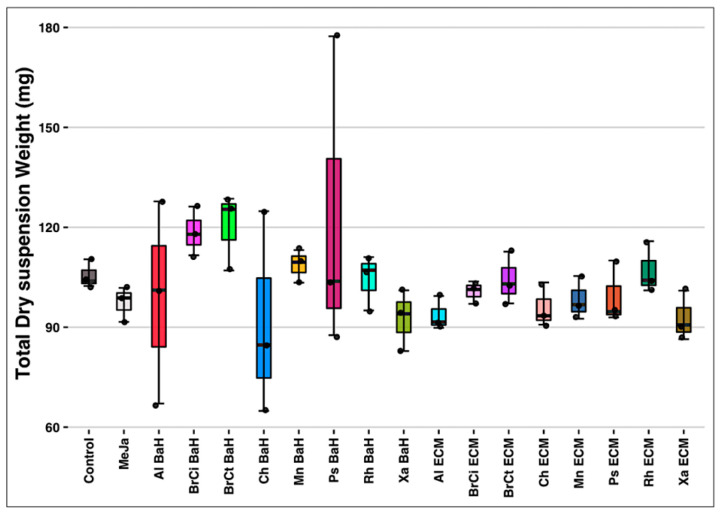
Total Dry Suspension Weight of *A. tinctoria* cells suspensions. BaH: bacteria homogenate; ECM: extracellular medium; Ch: *Chitinophaga* sp.; Xa: *Xanthomonas* sp.; Ps: *Pseudomonas* sp.; Mn: *Micromonospora* sp.; Al: *Allorhizobium* sp.; Rh: *Rhizobium* sp.; BrCi: *Brevibacillus* sp.; BrCt: *Brevibacterium* sp.

The Total Suspension Dry Weight (T-DW) was weighed at the Day 6 co-culture harvesting time. As shown in Figure 2, no significant impact was reported on biomass proliferation of *A. tinctoria* cell suspension with each of the eight bacterial endophytes co-culture (*p* = 0.469) compared to the control. The TDW of the control was observed at 105.553 ± 4.251 mg, while the TDW of the rest was recorded between 91.454 ± 30.549 mg and 120.345 ± 11.633 mg for *Chitinophaga* (Ch BaH) and *Brevibacterium* (BrCt BaH) spp. bacteria homogenates (at 0.04%), respectively. Despite only few monitored parameters, no biomass proliferation by the bacterial endophytes and no harmful impact on the plant cell culture was noticed.

### 3.2. A. tinctoria Metabolome Analysis Using UHPL-HRMS Untargeted Metabolomics

**Figure 3:**
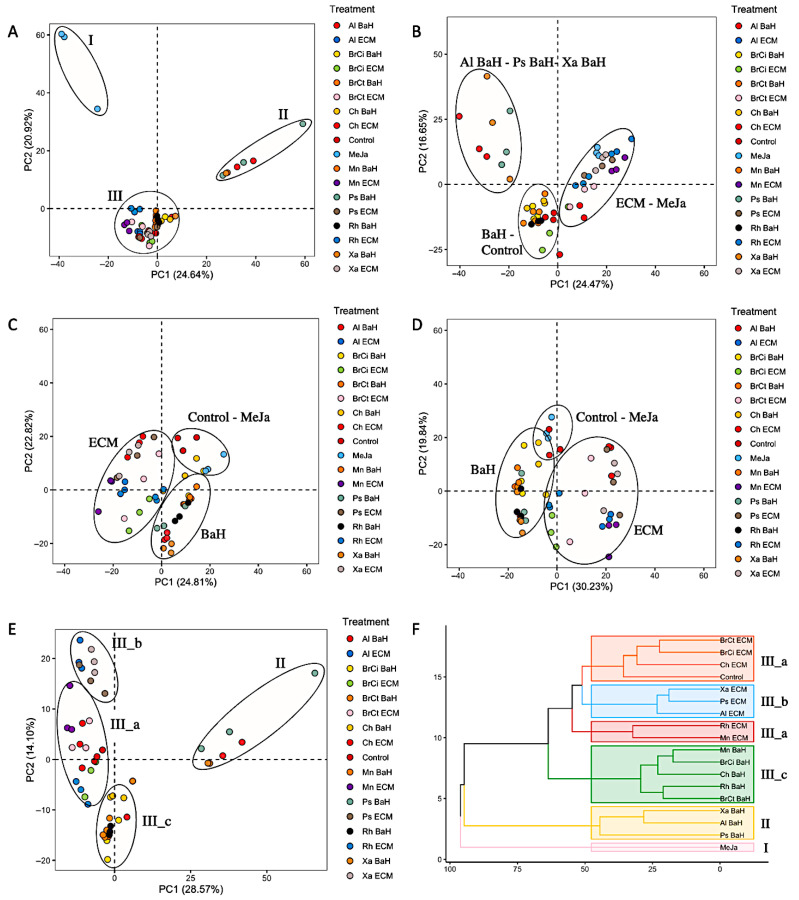
Untargeted metabolomics analysis *of A. tinctoria* suspensions associated with bacterial endophytes. PCA of EtoAc extract in (**A**) negative and (**B**) positive mode. PCA of EtOH in (**C**) negative and (**D**) positive mode. PCA of EtoAc extract in negative mode (**E**) excluding the methyl jasmonate condition. (**F**) Hierarchical clustering analysis of EtoAc extract in negative mode. BaH: Bacteria Homogenate; ECM: Extracellular medium; Ch: *Chitinophaga* sp.; Xa: *Xanthomonas* sp., Ps; *Pseudomonas* sp., Mn; *Micromonospora* sp., Al; *Allorhizobium* sp., Rh; *Rhizobium* sp., BrCi; *Brevibacillus* sp., BrCt; *Brevibacterium* sp.

The metabolite profiles of *A. tinctoria* cells suspension associated with bacterial endophytes components were assessed via UHPLC-HRMS mass spectrometry. Untargeted metabolomics based on UHPLC-HRMS analysis was favored, offering a meaningful approach for the comprehensive profiling and comparison of metabolites in biological systems [20]. Multivariate statistical analysis was performed to highlight significant differences in mass values between cultures (eighteen conditions, i.e., controls and associated bacterial component cultures). The features lists (i.e., a feature being an Rt associated with a *m*/*z*) of 54 samples (3 replicates per condition) containing 1237 and 1178 features for EtOAc extracts in negative and positive modes, respectively, and 595 and 776 features for EtOH extracts in negative and positive modes, respectively, were uploaded for principal component analysis (PCA) (Figure 3A–D). The score plots showed low variability among biological replicates, confirming a good repeatability of both the experimental and the analytical conditions. EtOAc extracts showed many more features than EtOH extracts, and they were therefore selected to highlight the bacterial endophytes metabolome impact on plant cell suspension.

PCA scores of EtOAc extracts in negative mode revealed a clear separation into three main groups (I–III) separated by principal components (PCs), which represented 45.72% of the total variance among samples of contributions to 24.64% by PC1 and 20.92% by PC2 (Figure 3A). The group I, located in the uppermost position of PC2, clustered the *A. tinctoria* cell suspension treated with the phytohormone, methyl jasmonate. The obvious distance of the group I from the other clusters confirmed the well-known high efficiency of methyl jasmonate to stimulate the biosynthesis of secondary metabolites in plant cell [21]. Interestingly, *A. tinctoria* cells suspension associated with *Xanthomonas* sp.-, *Pseudomonas* sp.-, and *Allorhizobium* sp.-bacteria homogenates, clustered in group II, were located in the uppermost position of PC1, indicating their significant impact on plant metabolism. Group I and II were largely distant, which highlight a clear differentiation on the secondary metabolites produced. In contrast to group I and II, group III, located in the center of the score plot clustered all the other bacterial endophytes—*A. tinctoria* cell suspension association and the control. This observation suggested that the bacteria endophytes components, except *Xanthomonas* sp.-, *Pseudomonas* sp.-, and *Allorhizobium* sp.-bacteria homogenates, did not induce a significant metabolome modification.

To go deeper, a principal component analysis was performed excluding cell suspensions treated with methyl jasmonate (Figure 3E). Similarly to the previous principal component analysis including all treatments, *A. tinctoria* cells suspension associated with *Xanthomonas* sp.-, *Pseudomonas* sp.-, and *Allorhizobium* sp.-bacteria homogenates were clustered at a distance from the others. Interestingly, group III was divided into three groups (IIIa–IIIc) (Figure 3E). Higher proximity in the uppermost position of PC2 was observed for the *A. tinctoria* cells suspension associated with *Xanthomonas* sp.-, *Pseudomonas* sp.-, and *Allorhizobium* sp.-extracellular medium (group III-b). Group III-c, located in the lowermost position of PC2, clustered the *A. tinctoria* cell suspension associated with bacteria homogenates of all bacterial endophytes except *Xanthomonas* sp.-, *Pseudomonas* sp.-, and *Allorhizobium* sp-. (group II). Finally, *A. tinctoria* cell suspension associated with extracellular medium of all bacterial endophytes except *Xanthomonas* sp.-, *Pseudomonas* sp.-, and *Allorhizobium* sp-. (group III-b) were pooled into the group III-a with the control.

This second principal component analysis provided critical additional information on the impact of bacterial endophytes on the plant cell suspension. *Xanthomonas* sp.-*, Pseudomonas* sp.-*, and Allorhizobium* sp. induced a modification of the metabolome different from the other endophytes both with bacteria homogenates and extracellular medium. Furthermore, being grouped separately, bacteria homogenates and extracellular medium affected the plant metabolism differently. In addition, *A. tinctoria* cell suspension associated with the extracellular medium of all bacterial endophytes except *Xanthomonas* sp.-, *Pseudomonas* sp.-, and *Allorhizobium* sp., did not induce a significant modification in the metabolome since they were clustered with the control. Similar clustering patterns were demonstrated by hierarchical clustering analysis, which confirmed the conclusions stated (Figure 3F).

### 3.3. Putative Identification of Induced Secondary Metabolites

The untargeted metabolomic analysis of *A. tinctoria* cells suspension associated with bacterial endophyte components revealed clear differences in their secondary metabolic profiles. The next step focused on gathering comprehensive information about the metabolites contributing to profile changes. Both positive and negative ion modes were examined, with the negative mode yielding more informative results (more features). The annotation process led to the tentative identification [22] of 32 compounds (Table 2).

Twenty-six phenolic compounds belonging to different chemical groups were tentatively characterized. Among them, eleven peaks were annotated as phenolic acid in Danshen (root or rhizome of *Salvia miltiorrhiza* Bunge) [23], made up of several monomers like danshensu and caffeic acid. Seven dimers, one trimer, and four tetramers, including two newly identified compounds, were identified. In addition, fifteen phenolic compounds derived from the geranylhydroquinone were detected such as one echinofuran, five hydroxyshikonofurans, and nine hydroxynaphthoquinones.

**Table 2:** Secondary metabolites identified in *A. tinctoria* cell suspensions stimulated with several endophytic bacteria materials by ^(−)^ ESI-HRMS and MS/MS analysis (ethyl acetate extract; negative mode).

No	Rt (min)	Proposed Phytochemicals	Precursor Ion [M−H]^−^	*m*/*z* Calcd.	Mass Error (ppm)	Chemical Formula	MS/MS Fragment Ions *m*/*z*	Reference
1	0.434	Gluconic acid	195.0503	195.0505	−1.0	C_6_H_12_O_7_	177.0793, 160.8910, 129.0183	[24]
2	0.547	p-hydroxybenzoic acid-O-glucoside	299.0758	299.0767	−3.0	C_13_H_16_O_8_	137.0228	[25]
3	0.890	3,4-dihydroxy benzene propionic acid	181.0490	181.0501	−6.1	C_9_H_10_O_4_	162.9812, 134.9859, 117.9341	[25]
4	1.053	p-hydroxybenzoic acid	137.0247	137.0239	5.8	C_7_H_6_O_3_	134.9865	[24]
5	1.830	3′-hydroxy-14-hydroxyshikonofuran H *	435.1660	435.1655	1.1	C_22_H_28_O_9_	273.1096, 255.1003, 145.9320	[26]
6	2.194	Prolithospermic acid	357.0609	357.0610	−0.3	C_18_H_14_O_8_	313.0686, 269.8307, 159.8919	[25]
7	2.344	Przewalskinic acid A	357.0595	357.0610	−4.2	C_18_H_14_O_8_	313.0686, 269.0803, 178.9772	[25]
8	3.377	Salvianolic acid G	339.0506	339.0505	0.3	C_18_H_12_O_7_	321.0781, 295.0620, 280.8622	[25]
9	3.378	Rabdosiin	717.1457	717.1456	0.1	C_36_H_30_O_16_	537.1082, 519.0881, 475.1022, 339.0496	[27]
10	3.405	Hydroxyshikinofuran A	333.1325	333.1338	−3.9	C_18_H_22_O_6_	273.1096, 255.0654	[26]
11	3.663	Salvianic acid A	197.0449	197.0450	−0.5	C_9_H_10_O_5_	179.0340,135.0443, 123.0437	[25]
12	3.663	Rosmarinic acid	359.0774	359.0767	1.9	C_18_H_16_O_8_	197.0448, 179.0340, 161.0240, 133.0288	[25]
13	3.834	Didehydrosalvianolic acid B	715.1301	715.1299	0.3	C_36_H_28_O_16_	337.0334, 319.1210, 293.8187	[28]
14	4.034	Salvianolic acid C	491.0968	491.0978	−2.0	C_26_H_20_O_10_	311.0545, 293.8111, 267.0631, 231.8548	[25]
15	4.090	Rosmarinic acid methyl ester	373.0919	373.0923	−1.1	C_19_H_18_O_8_	179.0339, 135.0445	[25]
16	4.298	Deoxyshikonofuran	257.1169	257.1178	−3.5	C_16_H_18_O_3_	173.0292, 159.8986, 116.9277	[26]
17	4.325	Caffeic acid	179.0345	179.0344	0.6	C_9_H_8_O_4_	135.8987	[25]
18	4.468	8′-decarboxy-rosmarinic acid *	313.0698	313.0712	−4.5	C_17_H_14_O_6_	179.0334, 161.0240, 133.0286, 123.0416	[29]
19	4.518	Lithospermidin C	345.0980	345.0974	1.7	C_18_H_18_O_7_	285.0727, 267.0626, 257.1180, 249.1110, 238.8891, 227.0691	[26]
20	4.589	Alkannin/Shikonin	287.0915	287.0919	−1.4	C_16_H_16_O_5_	218.8601, 190.9279, 189.9296, 173.0238, 161.0230	[26]
21	4.625	Arnebin V	289.1090	289.1076	4.8	C_16_H_18_O_5_	245.1179, 179.0702, 151.0398	[26]
22	4.654	Salvianolic acid F	313.0698	313.0712	−4.5	C_17_H_14_O_6_	269.0815, 203.0359, 161.0220, 133.0264, 123.0429	[30]
23	4.803	8‴-decarboxy-salvianolic B *	671.1400	671.1401	−0.1	C_35_H_28_O_14_	625.1328, 563.0319, 521.1053, 491.0969, 359.0759, 313.0711, 267.0643, 179.0338, 161.0237	[31]
24	4.853	Arnebin VI	347.1132	347.1131	0.3	C_18_H_20_O_7_	288.0997, 181.0493, 151.0395	[26]
25	5.052	Lithospermidin F	385.1277	385.1287	−2.6	C_21_H_22_O_7_	303.1212, 267.0644, 257.1188, 238.8907	[26]
26	5.352	8″-8‴-didecarboxy-salvianolic acid B *	625.1343	625.1346	−0.5	C_34_H_26_O_12_	339.1976, 313.0691, 238.8904, 149.0232, 133.0278, 116.9272	[31]
27	5.458	Hydroxyshikonofuran D/G	361.1636	361.1551	−4.2	C_20_H_26_O_6_	273.1121, 255.0974, 237.1086, 174.8651	[26]
28	5.765	O-Methyl-1′-deoxyalkannin	285.1118	285.1127	−3.2	C_17_H_18_O_4_	267.1491, 217.8558, 189.8500	[26]
29	5.808	Echinofuran B	255.1013	255.1021	−3.1	C_16_H_16_O_3_	173.0234, 159.0435, 116.9277, 100.9343	[26]
30	5.815	Deoxyalkannin (Arnebin VII)	271.0972	271.0970	0.7	C_16_H_16_O_4_	238.8911, 202.0260, 175.8447, 174.8634	[26]
31	6.107	Valerylshikonin	371.1488	371.1495	−1.9	C_21_H_24_O_6_	271.0966, 241.0714, 225.7941, 100.9306	[26]
32	6.357	Acetylalkannin	329.1003	329.1025	−6.7	C_18_H_18_O_6_	271.0941, 241.0745, 225.7967, 223.7977	[26]

* Reported for the first time in the literature.

**Figure 4:**
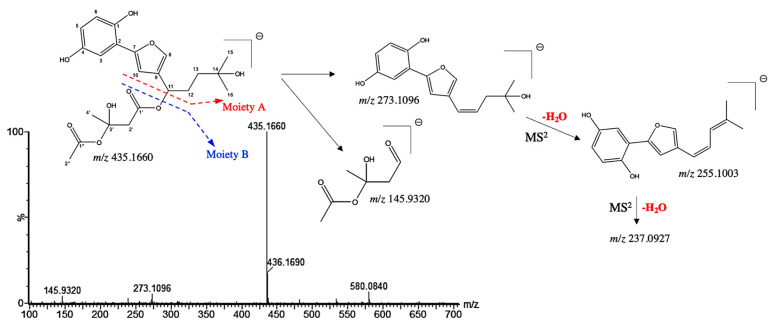
^(−)^ ESI-MS spectra and proposed MS^n^ fragmentation pathway of compound **5** identified by ESI-HRMS and MS/MS analysis.

Compound **5** presented a pseudo molecular [M−H]^−^ ion at *m*/*z* 435.1660 and shared a similar MS/MS fragmentation pattern with compounds **10** and **27**, tentatively assigned as hydroxyshikonofuran. Compounds **10** and **27** have been previously identified by Liao et al. by the extensive use of MS and MS/MS spectra [26]. Indeed, the MS/MS spectra of compound **5** shows ions at *m*/*z* 273.1096, resulting from a McLafferty rearrangement in moiety A (Figure 4). In addition, the characteristic second-generation product ions at *m*/*z* 255.1003 and at 237.0927 were obtained from the successive neutral loss of H_2_O of the moiety A (Figure 4). The RCOO^−^, obtained from moiety B at *m*/*z* 145.9320, was deduced from the hydroxyshikonofuran H described by Liao et al. Therefore, compound **5** was tentatively assigned as a 3′-hydroxy-14-hydroxyshikonofuran H with a chemical formula of C_22_H_28_O_9_.

**Figure 5:**
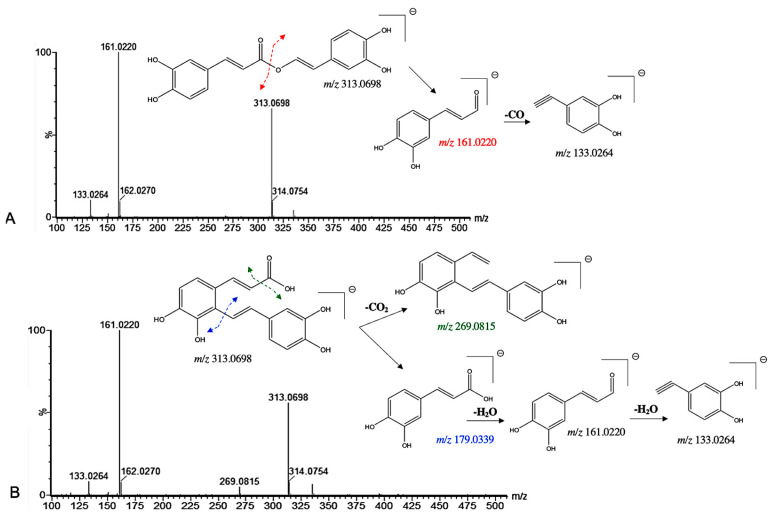
^(−)^ ESI-MS spectra and proposed MS^n^ fragmentation pathway of (**A**) compound **18** and (**B**) compound **22** identified by ESI-HRMS and MS/MS analysis.

Among the induced ions, two C_6_-C_3_ dimeric forms with a pseudo molecular [M−H]^−^ ion at *m*/*z* 313 were identified. Compound **18** presented a pseudo molecular [M−H]^−^ ion at *m*/*z* 313.0698 and prominent ion fragments at *m*/*z* 161.0220 and 133.0264 (Figure 5). Interestingly, a similar fragmentation pattern has been found for the rosmarinic acid [31]. However, a mass difference of 46 Da between compound **18** and the well-known rosmarinic acid was assigned. In particular, the lack of the fragment ion at *m*/*z* 197.0448 suggested the absence of the carboxyl group at C-8′ in the structure (Figure 5). Therefore, compound **18** was tentatively assigned as an 8′-decarboxy-rosmarinic acid with a chemical formula of C_17_H_14_O_6_. Similarly, compound **22** presented a pseudo molecular [M−H]^−^ ion at *m*/*z* 313.0698 and prominent ion fragments at *m*/*z* 161.0220 and 133.0264 (Figure 5). However, the presence of an additional fragment ion at *m*/*z* 269.0815 indicated that the structure may be different. Indeed, compound **22** has been previously identified by Grzegorczyk-Karolak et al. by the extensive use of MS and MS/MS spectra as salvianolic acid F [30].

**Figure 6:**
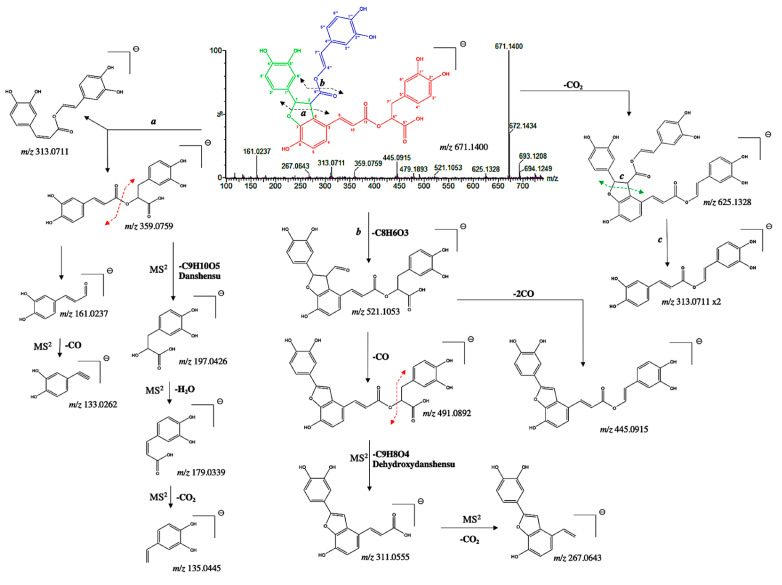
^(−)^ ESI-MS^n^ spectra and proposed MS^n^ fragmentation pathway of compound **23** identified by ESI-HRMS and MS/MS analysis.

In addition to the dimeric C_6_-C_3_ forms, two new tetramers were tentatively identified. Compound **23** presented a pseudo molecular [M−H]^−^ ion at *m*/*z* 671.1400 and prominent ion fragments at *m*/*z* 625.1328, 521.1053, 445.0915, 359.0759, 313.0711, 267.0643, and 161.0237 (Figure 6). The fragment ion at *m*/*z* 359.0773, resulting from the cleavage ***a*** of the hydroxybenzofuran ring, correspond to a rosmarinic acid unit [30]. The MS/MS spectra show the characteristic second-generation product ions at *m*/*z* 197.0426, 179.0339, and 135.0445, resulting from the three consecutive losses of danshensu (C_9_H_10_O_5_), H_2_O and CO_2_. Thus, this confirmed the existence of two C_6_-C_3_ units in the structure of compound **23** (part red; Figure 6). The fragment ion at *m*/*z* 521.1053 emerged from the cleavage ***b*** and produced two fragment ions at *m*/*z* 491.0892 and 445.0915, resulting from the loss of one or two CO, respectively. The MS/MS spectra show diagnostic fragments at *m*/*z* 311.0555 and 267.0643, resulting from the consecutive losses of dehydroxydanshensu (C_9_H_8_O_4_) and CO_2_. This clearly suggested the presence of a dihydroxy benzene hydroxybenzofuran on the structure, and thus, a third C_6_-C_3_ unit (part green; Figure 6). Interestingly, a similar fragmentation pattern has been found for the salvianolic acid B [31]. However, a mass difference of 46 Da with respect to salvianolic acid B was assigned. In particular, the diagnostic MS/MS fragments at *m*/*z* 625.1328 and 313.0711 suggested the absence of the carboxyl group at C-8‴ (part blue; Figure 6). Therefore, compound **23** was tentatively assigned as an 8‴-decarboxy-salvianolic acid B with a chemical formula of C_35_H_28_O_14_.

**Figure 7:**
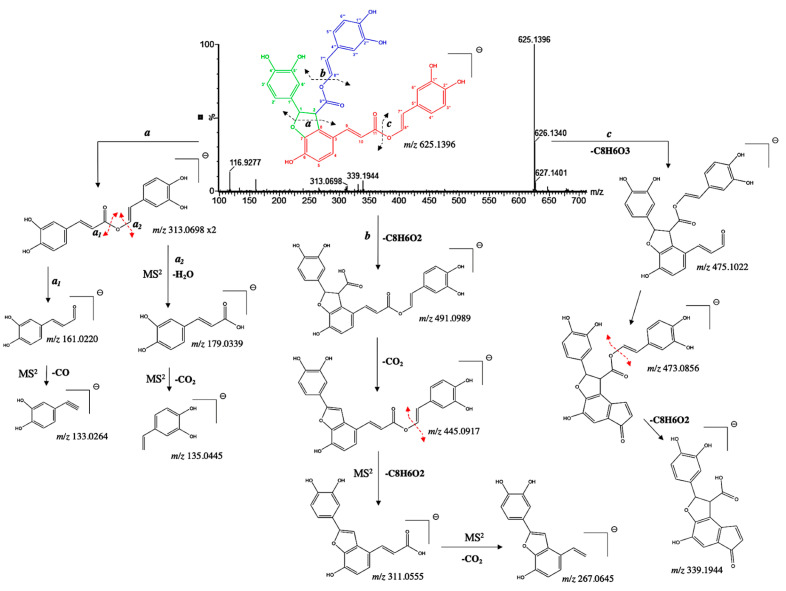
^(−)^ ESI-MS spectra and proposed MS^n^ fragmentation pathway of compound **26** identified by ESI-HRMS and MS/MS analysis.

Furthermore, compound **26** was identified as a new tetramer. It presented a pseudo molecular [M−H]^−^ ion at *m*/*z* 625.1328 and prominent ion fragments at *m*/*z* 491.0989, 445.0917, 313.0698, 179.0939, and 161.0220 (Figure 7). Interestingly, its pseudo molecular ion correspond to one of the fragment ions of compound **23**, corresponding to a neutral loss of a carboxyl group at position C-8″. Our suggestion was further confirmed by the diagnostic fragments at *m*/*z* 475.1022, 473.0856 and 339.1944, resulting in a McLafferty rearrangement [29]. Therefore, compound **26** was tentatively assigned as an 8″-8‴-didecarboxy-salvianolic acid B with a chemical formula of C_34_H_26_O_12_.

**Figure 8:**
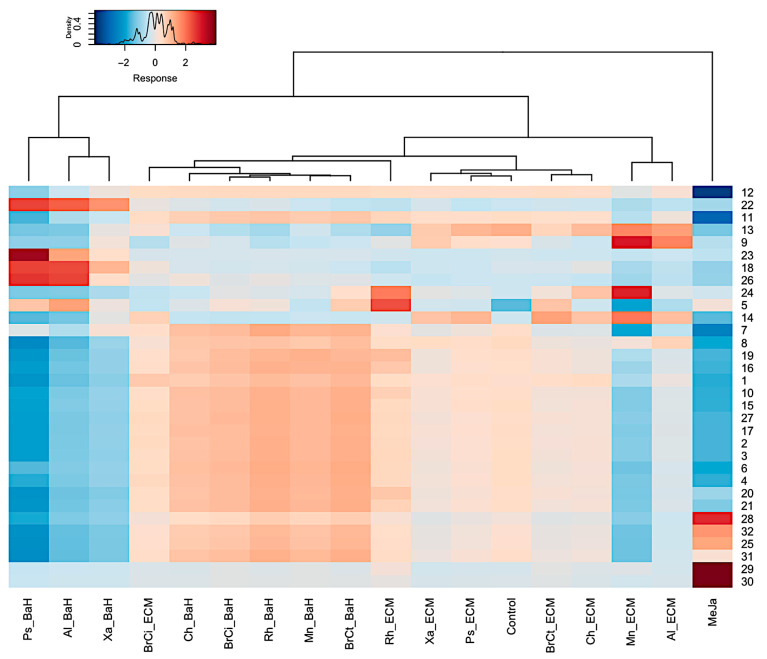
Heatmap with hierarchical clustering analysis (Euclidean distance) of the 32 metabolites identified by ^(−)^ ESI-HRMS and MS/MS analysis extracted using ethyl acetate. Each row represents a single metabolite and each column represents one condition of culture. Red and blue indicate an increase and decrease in metabolites content, respectively (see color scale above the heatmap). BaH: Bacteria Homogenate; ECM: Extracellular medium; Ch: *Chitinophaga* sp.; Xa: *Xanthomonas* sp.; Ps: *Pseudomonas* sp.; Mn: *Micromonospora* sp.; Al: *Allorhizobium* sp.; Rh: *Rhizobium* sp.; BrCi: *Brevibacillus* sp.; BrCt: *Brevibacterium* sp.; MeJa: methyl jasmonate.

After the dereplication process, the cluster separation observed in the PCA was confirmed by a heatmap (Figure 8). The heatmap was generated according to the abundance of the 32 secondary metabolites identified, representing differential expression of the phenolic acids in Danshen (e.g., monomers, dimers, trimer and tetramers) and the phenolic compounds derived from the geranylhydroquinone as echinofuran, hydroxyshikonofurans and hydroxynaphthoquinones. Among the phenolic compounds derived from geranylhydroquinone, a significantly higher number of hydroxynaphthoquinones were upregulated in response to methyl jasmonate, in particular, deoxyalkannin (**30**), *O*-methyl-1′-deoxyalkannin (**28**), valerylshikonin (**31**), and acetylalkannin (**32**). In contrast, phenolic acids in Danshen contents such as the newly identified dimer and tetramers (i.e., 8′-decarboxy-rosmarinic acid (**18**), 8‴ decarboxy-salvianolic B (**23**), 8″-8‴-didecarboxy-salvianolic acid B (**26**)) were upregulated in response to *Xanthomonas* sp.-, *Pseudomonas* sp.-, and *Allorhizobium* sp.-bacteria homogenates. Similarly, phenolic acids in Danshen like the trimer salvianolic acid C (**14**) and the two tetramers rabdosiin (**9**) and didehydrosalvianolic acid B (**13**) were upregulated in response to bacterial components as *Micromonospora* sp.-, and *Allorhizobium* sp.-extracellular medium. Interestingly, the compound **5** tentatively characterized a 3′-hydroxy-14-hydroxyshikonofuran H was the most upregulated in response to *Rhizobium* sp.-extracellular medium. In addition, biosynthesis intermediates of phenolic compounds were accumulated in the extracts of *A. tinctoria* associated with bacteria homogenates, which may suggest harvesting too early due to a slower biosynthetic pathway activating effect.

The heatmap showed a similar clustering than with PCA, confirming the modification of the metabolism engendered by the phytohormone, methyl jasmonate and by the bacterial endophyte components.

## 4. Discussion

A complex assortment of beneficial micro-organisms develops in the different organs of the plant, e.g., in seeds, leaves, flowers, or roots [2]. Bacterial endophytes are among those microorganisms improving plant nutrition and growth, stimulating defense mechanisms, and impacting the up- and downregulation of specific secondary metabolites in plants [5]. Colonization of plant tissues by bacterial endophytes leads to qualitative and quantitative changes in secondary metabolites, some of which may be commercially valuable.

To address this question, co-culture experiments were conducted in vitro to investigate the role of bacterial endophytes in bioactive secondary metabolites production of the medicinal plant *A. tinctoria*. An untargeted UHPLC-HRMS metabolomics approach combined with multivariate data analysis enabled to provide a broad picture of the *A. tinctoria* metabolic profile changes as a result of the presence of bacterial endophytes in the culture.

### 4.1. Suitability of In Vitro Bacterial Endophyte–Plant Co-Culture for the Production of Bioactive Secondary Metabolites

Biotechnological advancements provide us the opportunity to make use of plant cells of economically important plants by growing them under in vitro specific conditions to obtain the desired compounds [32]. Further, these strategies can recreate the plant–microorganism interactions that take place in their natural habitat. In particular, the addition of bacterial endophyte components in the plant cell culture may mimic the intimate molecular interactions established between the plants and their associated microorganisms and stimulate a targeted synthesis of secondary metabolites. Hence, in vitro production of high-value metabolites by plant cells could be helpful in understanding the biosynthesis of these compounds.

In vitro cultures of *Alkanna* species have been already reported [33,34,35]. Furthermore, the stimulation of HNQs production by phytohormones including jasmonate analogues and exogenous polysaccharides in suspension cultures of *A. tinctoria* was demonstrated in a previous study [36,37]. In our study, the bacterial endophytes from roots of *A. tinctoria* promoted the biosynthesis of HNQs and other compounds of interest. Interestingly, in comparison to other plant organs, root bacterial microbiota often contains the highest diversity of microorganisms [3].

*A. tinctoria* cells suspension prosperity was clearly observed in the presence and absence of the bacterial endophyte components in the culture. This was noticed by no significant differences in the total dry suspension weight between all conditions. Thus, this confirmed that highly specialized symbioses require tight coordination of physiology, structure, and life cycles between the partner organisms, and the resulting partnership benefits both species [4].

In addition, plant cells were strictly grown with the same amount of nutrients throughout the experiment, and a nearly identical suspension weight was observed, resulting in no noticeable impact on plant growth. This could be attributed to the high cell concentration per volume at the inoculum (200 g/L). Indeed, our investigation aimed at studying the impact of the plant secondary metabolism in response to bacterial endophytes, the inoculation was carried out on a plant cell suspension at the peak of its cell growth. The energy required for plant growth limits the production of secondary metabolites requiring equally high energy.

The in vitro co-culture experiment appeared to be well-suited for observing metabolic changes without inducing any negative effects on the plant cells.

### 4.2. Impact of Bacterial Endophytes on Secondary Metabolism of A. tinctoria In Vitro Culture System

The selection of eight bacterial endophytes isolated from the roots of wild *A. tinctoria* was tested in vitro for their effect on secondary metabolite production. It has been demonstrated that three of them, *Allorhizobium* sp., *Xanthomonas* sp., and *Pseudomonas* sp., induced an accumulation of phenolic compounds in the plant cells. These findings support the conclusions of the studies by Rat et al. and Alonso et al., showing the induction of secondary metabolite biosynthetic pathways of Boraginaceae by *Xanthomonas* sp. *and Pseudomonas* sp. *A. tinctoria* endophytes [15,38]. Studied widely in the literature, *Bacillus* sp., *Pseudomonas* sp., and *Paenibacillus* sp. were identified to influence the growth, stress resistance, and metabolism of medicinal plants [4].

To go deeper into the investigation, the most impactful modification of the metabolome was observed by the bacteria homogenates of *Allorhizobium* sp., *Xanthomonas* sp., and *Pseudomonas* sp., which can be explained by the presence of lipopolysaccharides (or lipids) in bacteria membrane elements. These last were reported as plant elicitors from certain bacteria such as *Pseudomonas* sp. and *Xanthomonas* sp. [2]. Besides bacteria homogenates, a slight variation in secondary metabolite production by the extracellular media was also observed during this study. Studies demonstrated that bacteria released elicitors that act on plant hosts and induce secondary metabolites synthesis [2].

**Figure 9:**
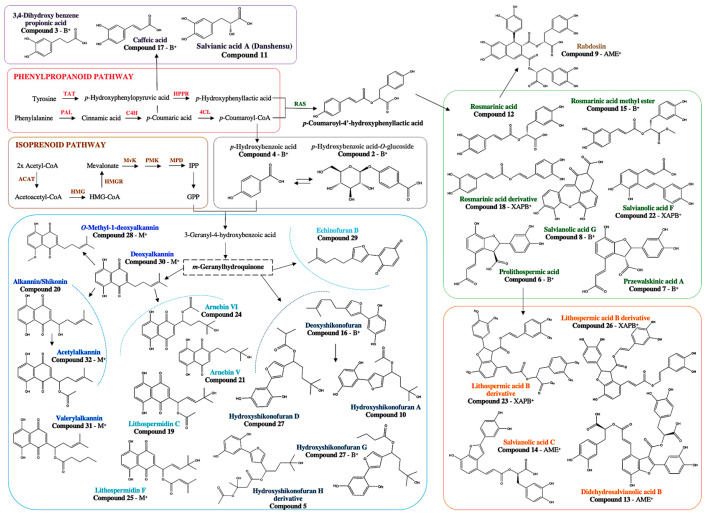
Schematic representation of the main SMs influenced by the endophytic bacteria in relation to different biosynthetic pathways. (PAL, phenylalanine ammonia-lyase; TAT, tyrosine aminotransferase; C4H, cinnamic acid-4-hydrolase; HPPR, hydroxyphenylpyruvate reductase; 4CL, 4-courmaric acid coenzyme A-ligase; RAS, rosmarinic acid synthase; ACAT, acetyl-CoA acetyl transferase; HMG, 3-hydroxy-3-methylglutaryl-CoA synthase; HMGR, 3-hydroxy-3-methylglutaryl-CoA reductase; MvK, mevalonate kinase; PMK, phosphomevalonate kinase; MPD, mevalonate-PP decarboxylase).

To gain insights into the induction mechanisms of secondary metabolites, the results from multivariate statistical analysis and the dereplication process (Figure 9) clearly show that different biosynthetic pathways were modulated depending on the culture conditions.

Inoculation with the bacteria homogenates of *Allorhizobium* sp., *Xanthomonas* sp., and *Pseudomonas* sp. was associated with a higher production of phenolic acid in Danshen, lithospermic acid B derivatives, compared to the methyl jasmonate treatment. As phenolic acid in Danshen and phenolic compounds derived from the geranylhydroquinone, such as hydroxynaphthoquinones, share a metabolic precursor (*p*-coumaric acid), it is possible that a strong induction of lithospermic acid B derivatives led to the side production of geranyl hydroquinone derivatives. These compounds were putatively derived from rosmarinic acid, which is an ester of caffeic acid and 3,4-dihydroxyphenyllactic acid, the most frequently occurring caffeic acid esters in the plant kingdom besides chlorogenic acid [39].

Interestingly, the effect of other bacteria homogenates (i.e., *Chitinophaga* sp., *Brevibacterium* sp., *Brevibacillus* sp., *Micromonospora* sp.) was less targeted towards the induction of particular metabolites but instead activated the plant’s general metabolic response. Conversely to these treatments, the inoculation with *Rhizobium* sp. extracellular medium involved mostly the induction of compound 5, 3′ hydroxy-14-hydroxyshikonofuran H. This highlighted the ability of the bacteria to stimulate the production of specific metabolites.

## 5. Conclusions

Overall, in this study, we developed an in vitro plant cell cultivation system that enabled efficient screening of bacterial endophytes for their ability to induce secondary metabolite production, offering new opportunities towards the application of microorganisms to improve the production of valuable phytochemicals in plants. Through an UHPLC-HRMS-based hierarchical clustering analysis and principal component analysis, significant modifications in SMs production caused by bacterial endophytes were highlighted in *A. tinctoria*. Dimer and tetramers (i.e., 8′-decarboxy-rosmarinic acid (**18**), 8‴decarboxy-salvianolic B (**23**), 8″-8‴-didecarboxy-salvianolic acid B (**26**)), and 3′hydroxy-14-hydroxyshikonofuran H (**5**) were putatively identified for the first time. Our results revealed specific regulation patterns in the production of secondary metabolites of *A. tinctoria* cell culture and pave the road for further studies on this plant with interesting perspectives for industry and pharma. Thus, a targeted approach with selected microbial inoculants applied under highly controlled conditions may be of interest to increase or stimulate the production of specific high-value metabolites.

## Figures and Tables

**Table 1:** Bacterial endophytes isolated from wild *A. tinctoria* roots. * HNQs inducers.

Bacterial Phylum	Bacterial Genus	Strain	Bacterial Material Plant Modulator Type	Concentration	Code
Bacteroidota	*Chitinophaga* sp. *	*R-73072*	Bacteria Homogenate	0.04%	Ch BaH
			Extracellular medium	4%	Ch ECM
Pseudomonadota (class Gammaproteobacteria)	*Xanthomonas* sp. *	*R-73098*	Bacteria Homogenate	0.04%	Xa BaH
			Extracellular medium	4%	Xa ECM
	*Pseudomonas* sp. *	*R-71838*	Bacteria Homogenate	0.04%	Ps BaH
			Extracellular medium	4%	Ps ECM
Actinomycetota	*Micromonospora* sp. *	*R-75348*	Bacteria Homogenate	0.04%	Mn BaH
			Extracellular medium	4%	Mn ECM
Pseudomonadota (class Alphaproteobacteria	*Allorhizobium* sp. *	*R-72379*	Bacteria Homogenate	0.04%	Al BaH
			Extracellular medium	4%	Al ECM
	*Rhizobium* sp.	*R-72160*	Bacteria Homogenate	0.04%	Rh BaH
			Extracellular medium	4%	Rh ECM
Bacillota	*Brevibacillus* sp.	*R-71971*	Bacteria Homogenate	0.04%	BrCi BaH
			Extracellular medium	4%	BrCi ECM
	*Brevibacterium* sp.	*R-71875*	Bacteria Homogenate	0.04%	BrCt BaH
			Extracellular medium	4%	BrCt ECM

* HNQs inducers; bacterial endophytes were identified and characterized by Rat et al. [15].

## Data Availability

All raw data are available at Zenodo: https://doi.org/10.5281/zenodo.15514400 (accessed on 26 May 2025).

## Abbreviations

4CL	4-courmaric acid coenzyme A-ligase
ACAT	Acetyl-CoA acetyl transferase
ACN	Acetonitrile
Al	*Allorhizobium* sp.
BaH	Biomass homogenate
BAP	6-benzylaminopurine
BrCi	*Brevibacillus* sp.
BrCt	*Brevibacterium* sp.
C4M	Cinnamic acid-4-hydrolase
Ch	*Chitinophaga* sp.
DMSO	Dimethyl sulfoxide
ECM	Bacterial endophyte culture supernatant
EtoAC	Ethyl acetate
EtOH abs	Ethanol absolute
HMGR	3-hydroxy-3-methylglutaryl-CoAsynthase
HNQ	Hydroxynaphthoquinones
HPPR	Hydroxyphenylpyruvate reductase
IAA	Indole-3-acetic acid
LC/MS	Liquid chromatography/mass spectrometry
MeOH	Methanol
Mn	*Micromonospora* sp.
MPD	Mevalonate-PP decarboxylase
MS/MS	Mass spectrometry/mass spectrometry
MvK	Mevalonate kinase
PAL	Phenylalanine ammonia-lyase
PCA	Principal component analysis
PMK	Phosphomevalonate kinase
Ps	*Pseudomonas* sp.
RAS	Rosmarinic acid synthase
Rh	*Rhizobium* sp.
SM	Secondary metabolite
T-DW	Total suspension dry weight
TAT	Tyrosine aminotransferase
UHPLC-HRMS	Ultra-high-performance liquid chromatography high-resolution mass spectrometry
Xa	*Xanthomonas* sp.
